# The Outcome of Modified Mini-Open Brostrom Gould Ankle Surgery on Chronic Ankle Instability

**DOI:** 10.7759/cureus.40656

**Published:** 2023-06-19

**Authors:** Amarinda Tan, Mark Chan, Charles Kon Kam King, Darshana Chandrakumara, Raj Socklingam

**Affiliations:** 1 Orthopedic Surgery, Yong Loo Lin School of Medicine, Singapore, SGP; 2 Orthopaedic Surgery, Changi General Hospital, Singapore, SGP

**Keywords:** ankle and foot, orthopaedic surgery, anterior talofibular ligament, lateral ankle ligament instability, chronic ankle instability, brostrom technique

## Abstract

Introduction

Modified Brostrom-Gould surgery (MBG) aims to repair the lateral ligaments of the ankle in patients with ligamentous laxity and chronic instability. Brostrom-Gould surgery-the Brostrom technique associated with Gould augmentation-is currently the gold standard surgical option for chronic ankle instability worldwide. Chronic lateral ankle instability caused by lateral ankle sprains is one of the most common sports-related injuries, and Brostrom-Gould surgery is commonly recommended as the operative treatment. While arthroscopic surgery is becoming the more heavily favored approach of choice, open Brostrom-Gould surgery is still pertinent for patients for whom arthroscopic repair is unsuitable.

Aim

This paper discusses a modified mini-open approach of the open Brostrom-Gould surgery with a smaller incision (1.5 cm) and aims to study the outcomes of this modified approach on patients' post-operative pain, stability, and functional outcome.

Methods

Forty-two patients were followed up for a mean of 2.6 years after undergoing modified mini-open Brostrom-Gould surgery. The Visual Analog Scale (VAS), the Foot and Ankle Outcome Score (FAOS), and Karlsson scores were used to monitor their post-operative recovery. The Wilcoxon signed-rank test and the SPSS Statistics (v.28.0.1) software were used for data management and analytics.

Results

The results showed a mean Karlsson score of 83.4, a mean FAOS score of 69.7, and a mean VAS score of 1.33. These results are comparable to studies conducted on conventional open Brostrom-Gould repair.

Conclusion

The modified mini-open Brostrom Gould provides a favorable functional outcome with a reduction in pain and suggests no decrease in efficacy with the modified approach. This is coupled with the added advantages of a smaller wound, better wound healing outcomes, and availability to patients not suited to arthroscopic repair.

## Introduction

Ankle sprains comprise up to 75% of ankle injuries involving the lateral ligamentous complex, often leading to chronic ankle instability [1]. The first-line treatment usually involves conservative methods, such as functional rehabilitation therapy, pharmacological anti-inflammatories, and physiotherapy. However, most patients with chronic ankle instability cannot return to their activity levels prior to their injury, despite conservative treatment [2]. Up to 34% of acute ankle sprains can lead to the development of chronic ankle instability, as suggested by recurrent ankle sprains [3]. Hence, it is imperative to prevent the progression of chronic ankle instability and the associated complications. Up to 70% of ankle osteoarthritis cases may be associated with a previous ankle injury [4]. Long-standing lateral ligament instability of the ankle results in uneven loading of the medial joint space, which, in turn, can lead to degenerative arthritis. In one clinical study, 80% of patients with severe degenerative arthritis subsequently required total ankle replacements [5]. Chronic ankle instability caused by ligamentous injury commonly results in severe sprains; approximately 15% are recurrent [6].

Surgery is usually indicated if the initial conservative treatment is refractory for 3-6 months or if there are signs and symptoms suggestive of chronic ankle instability, such as recurrent ankle sprains, or if instability is observed on imaging modalities [4]. While acute lateral ligament injuries usually have a good prognosis with conservative therapy involving rest, ice packs, nonsteroid anti-inflammatory drugs (NSAIDs), bandaging the limb followed by the use of a splint, early motion, and physical therapy, when these injuries are not properly managed, the main complication occurring in ankle lateral ligament injuries is the development of chronic ankle instability. The development of chronic ankle instability from acute injuries due to failure of conservative management would be multifactorial, involving poor compliance to medications and physiotherapy, insufficient rest, and poor rehabilitative potential [7]. Modified Brostrom-Gould surgery (MBG) is a procedure for lateral ankle instability that repairs the lateral ankle ligaments, such as the anterior talofibular ligament (ATFL) and the calcaneofibular ligament (CFL). The rupture of these ligaments typically results from ankle sprains, chronic lateral ankle instability, or severe acute ankle ligament injuries and can lead to chronic sprains, cartilage damage, osteophyte formation, peroneal injuries, and pain [8]. Anatomical repair is achieved through a suture anchor technique, where anchors are placed into the cortex of the fibula. In recent years, numerous clinical studies have been done to measure the outcomes of conventional open MBG. There have also been increasing numbers of studies to compare the outcomes of open versus arthroscopic MBG [8-17]. However, few studies have discussed the outcomes of a mini-open approach, which may benefit patients who may not be suitable for arthroscopic surgery. This study focuses on the modified mini-open Brostrom-Gould surgery (MMBG). 

Given that ankle sprains and the resultant chronic ankle instability have significant public health implications, it is of significant benefit to study the methods of repair for ankle sprains to further understand their outcomes. This study aims to use different scoring systems, i.e., Karlsson scores, the Foot and Ankle Outcome Score (FAOS), and the Visual Analog Scale (VAS), to assess if the MMBG allows for an improvement in post-operative pain, stability, and functional ability of the affected ankle.

## Materials and methods

Surgical Technique

**Figure 1:**
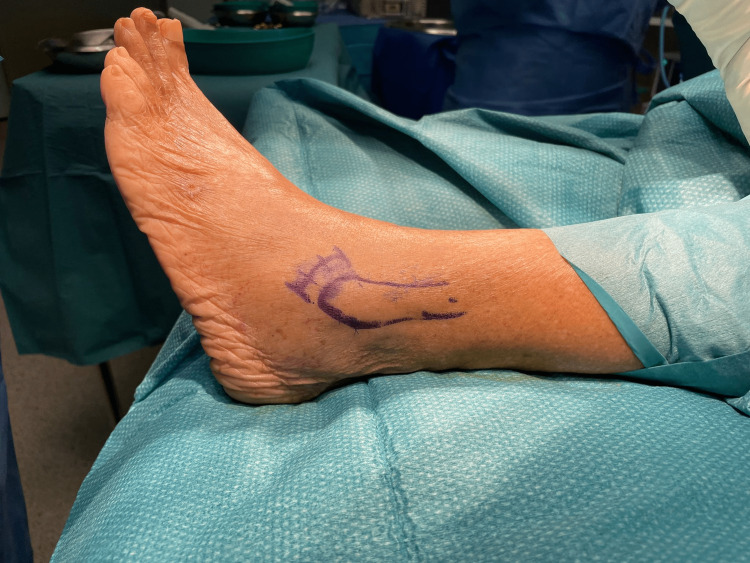
Marking for incision of modified mini-open Brostrom Gould surgery

With the patient in a supine position, an inverted J-shaped curvilinear incision is made over the distal fibula, parallel and 3-5 mm proximal to the distal edge, anterior to the lateral malleoli. Figure 1 shows the incision markings for the MMBG. Compared to MBG's extensile incision, this J-shaped curvilinear incision of just 1 to 2 cm allows for a smaller incision while ensuring access to both the ATFL and CFL.

**Figure 2:**
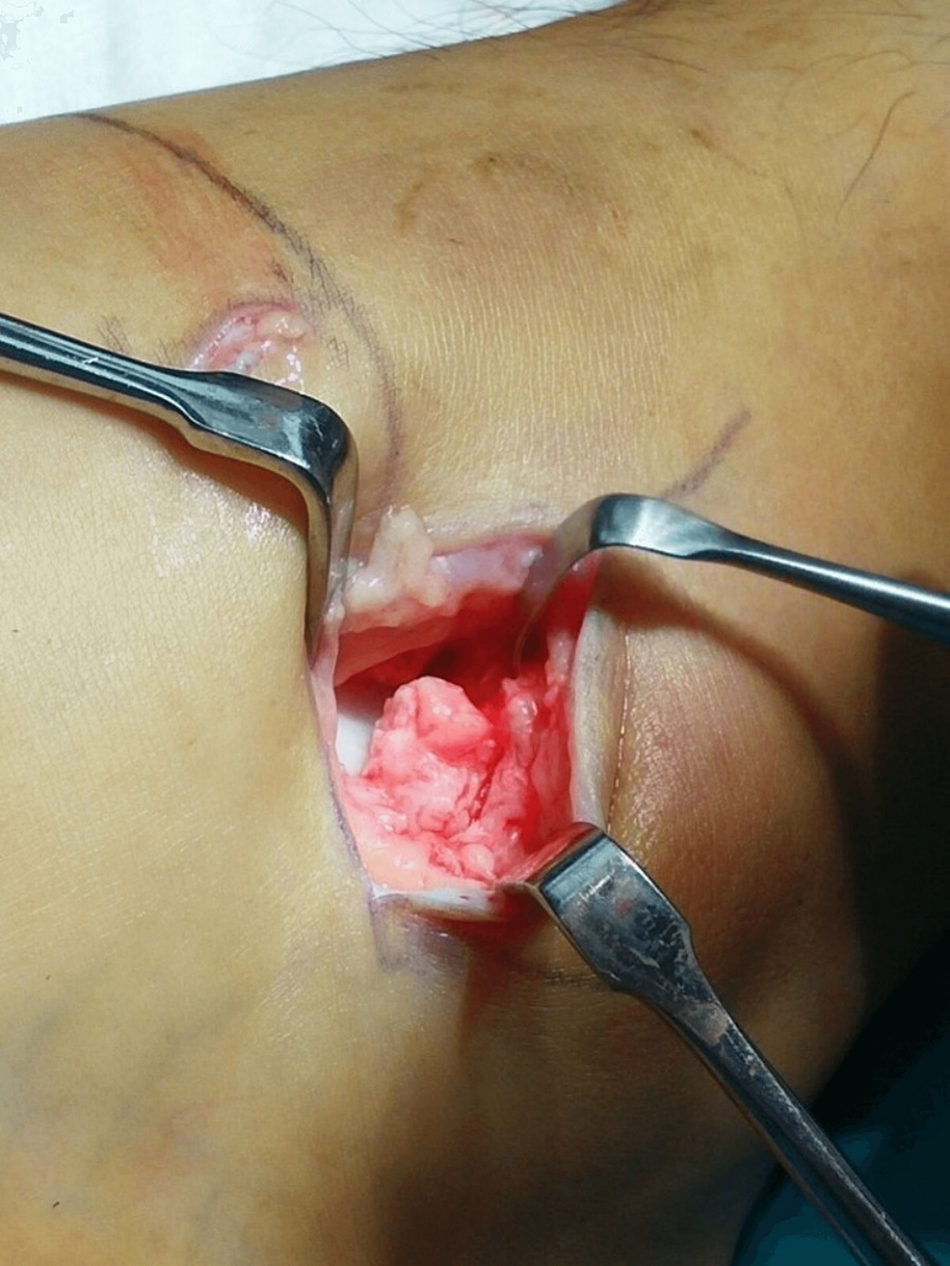
Incision over the distal fibular

**Figure 3:**
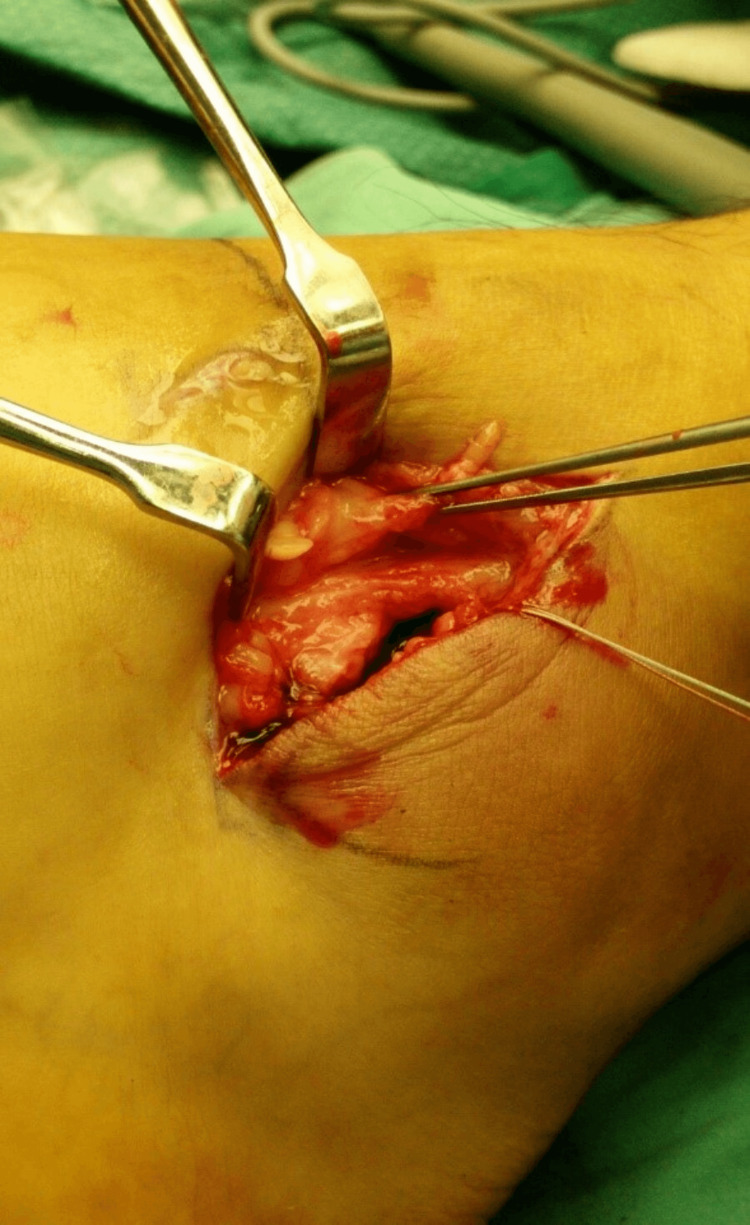
Exposed retinaculum

The superficial peroneal retinaculum is dissected from the underlying capsule and ligaments. The capsule and lateral ligaments (the ATFL and the CFL) are detached from the fibula by sharp dissection, as shown in Figures 2-3. Care is taken to leave a cuff of capsule and ligaments by making the incision a few millimeters proximal to the fibular edge. Any osteophytes or loose bone fragments are removed.

The bone is prepared for anchor insertion using two suture anchors. In this study, the participants were operated on using either second-generation Smith and Nephew SUTUREFIX 1.7 mm anchors or JuggerKnot® all-suture anchors. The bone is pre-drilled, and the first anchor is inserted. The anchor is fully seated when inserted into the laser line on the driver shaft. The classic Brostrom-Gould technique does not employ suture anchors but utilizes suture imbrication and repair of the ATFL and the CFL in a pants-over-vest fashion with subsequent inferior retinacular reinforcement. This MBG involves suture anchors as described.

The suture with needles is passed through the ATFL, and the capsule is advanced into the distal fibula. The ATFL is repaired to the footprint, using two sutures from inferior to superior. First, the sutures are passed through the ATFL ligament. Next, they are passed underneath the periosteum, exiting superficially. The second anchor reinforces this once the extensor retinaculum is advanced over the ATFL repair and proximally sutured to the remaining periosteal flap.

During the first two weeks of post-operative rehabilitation, the patients are advised for full weight bearing with a stirrup brace as tolerated, allowing for an active range of motion. The patients are advised to plantarflex and dorsiflex only as tolerated. At six weeks post-operation, the patients are advised for full weight bearing without the stirrup brace. A crutch may be offered to normalize gait patterns. The aim at this point is to return to function and to allow for inversion and eversion as tolerated. At 12 weeks post-operation, patients are cleared to return to running. At four months post-surgery, patients are cleared to return to sports training. By six months post-operation, patients should have returned to sports and be aimed for discharge.

Study design

A retrospective study was conducted on 47 participants. The participants were recruited through a public hospital in Singapore, where they underwent an MMBG by a single surgeon between November 2017 and July 2019. This would mean a 2-4 years post-operation duration, with a mean of 2.6 years. The inclusion criterion was any patient who received the MMBG. Post-operatively, the patients were asked to fill out the patient-reported outcome measures according to different scoring systems, such as pain scores and activity levels before and after surgery. Other information gathered included any recurrent ankle sprains and time spent exercising. Of the 47 participants, five were uncontactable. For the purposes of this study, the pre-operative data for these five participants were not taken into account, and we focused only on the 42 participants who were followed up. The subsequent parts of this study will reflect the data collected from the 42 participants only. This study was approved by the institution IRB 2017/2947.

Data collection

First, data on the history of the participant's presenting complaint that required the surgery, such as the date of first sprain, the total number of ankle sprains, and personal past medical history, were taken. Other information collected included the participant's occupation, whether the injury was a work injury, and any previous injuries to the affected ankle.

Second, it was essential to collect data on the participant's activity levels and functional ability of the ankle prior to the surgery, including pain score, level of instability, and ability to walk, run, and climb stairs. Additionally, data on support required for the ankle were collected, such as using ankle guards or kinesiology tape. The participant was then awarded points according to the different scoring systems based on their responses.

Third, information on the functional ability of the affected ankle was collected. This included current pain scores and any remaining instability, stiffness, or decreased range of motion. This was then used to generate Karlsson scores and the FAOS. In addition, data on post-operative ankle sprains and whether any revision surgery was required were collected.

Background demographics

Among the 42 participants, ages at surgery ranged from 19-61 years old, with 31 males and 11 females.

Statistical analysis

An individual statistician conducted statistical analysis at a public hospital in Singapore. The data analysis was carried out using the SPSS Statistics (v.28.0.1) software; P-values were calculated and extracted using the Wilcoxon signed-rank test. The minimum, maximum, mean, and median scores were calculated. Statistical analysis will be conducted using descriptive statistics for continuous variables. Each category of scores, Karlsson scores, and the FAOS were analyzed and will be discussed separately. Variables were considered significant if P-values were less than 0.05.

## Results

A total of 42 patients were included, 31 males and 11 females ranging from 19 to 61 years of age, with an average of 34.6 years. A full breakdown of the individual scoring systems can be found below.

VAS pain score comparison

**Figure 4:**
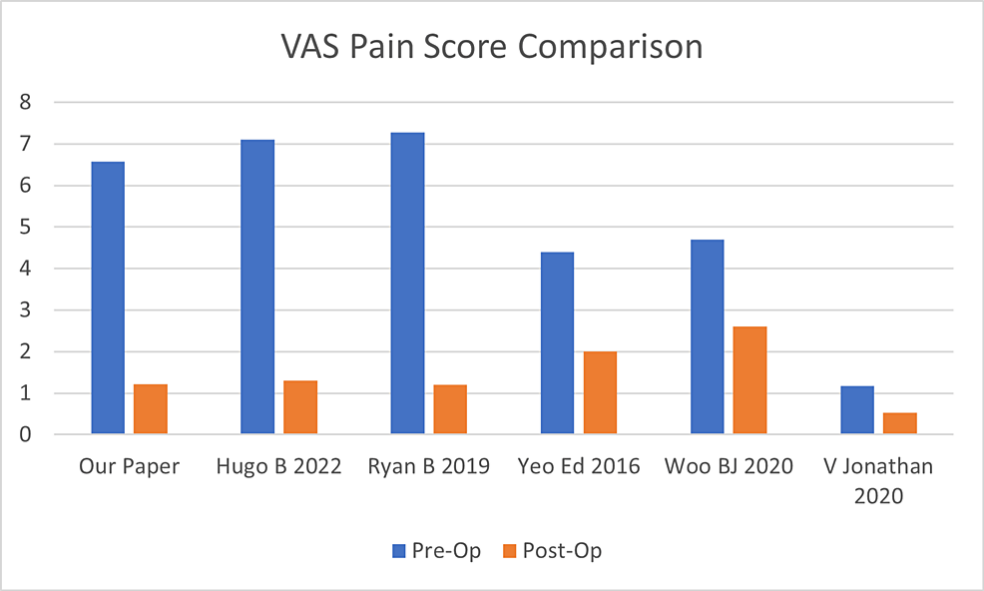
VAS pain score comparison VAS: Visual Analog Scale

On analysis, the average pre-operative pain VAS score (Table 1) was 6.57 and reduced to an average of 1.33 2.6 years post-operation. The pre-operative VAS score was reported in five studies (Figure 4), with 164 patients treated with open repair [9-13]. The average pre-operative VAS score was 5.89. The average postoperative VAS score reported in these studies was 1.53. This corresponds closely to the results collected in our study.

**Table 1:** Pre-operative and post-operative VAS scores and associated P-values. VAS: Visual Analog Scale

VAS score	Pre-operative VAS score	Post-operative VAS score	P-value
Pain	6.6	1.3	<0.001

Karlsson score comparison

**Figure 5:**
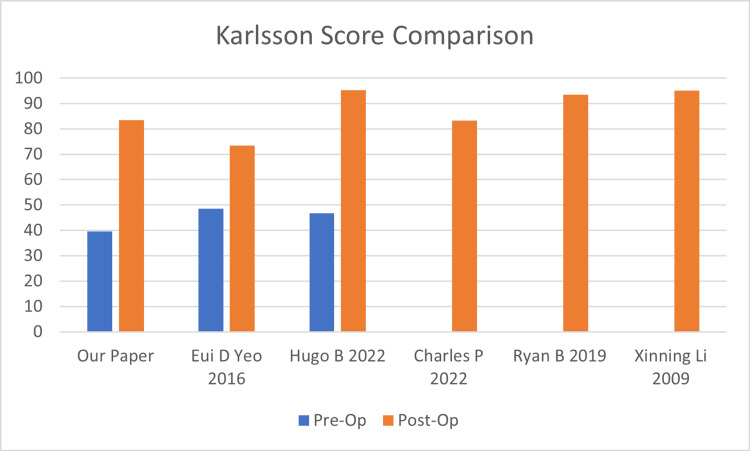
Karlsson score comparison

The pre-operative mean Karlsson score (Table 2) was 39.6 (minimum 5, maximum 80). Post-operatively, there were significant improvements in the various scores. The mean Karlsson score (Table 2) was 83.4 post-operatively. The pre-operative Karlsson score was reported in two studies, with 78 patients treated with open repair. The average Karlsson score was 47.7. The post-operative Karlsson score was reported in five studies (Figure 5), with 243 patients averaging 88.1 [9-11,14-15]. This corresponds closely to the results collected in our study.

**Table 2:** Pre-operative and post-operative Karlsson scores broken down into specific components and associated P-values.

Karlsson ankle function score	Pre-operative Karlsson score	Post-operative Karlsson score	P-value
Swelling	4.6	9.8	<0.001
Instability	4.8	14.2	<0.001
Stiffness	2.3	3.7	<0.001
Stair climbing	5.5	9.3	<0.001
Running	5	9.3	<0.001
Work activities	7.6	14.3	<0.001
Support	1.8	4.5	<0.001

FAOS score comparison

**Figure 6:**
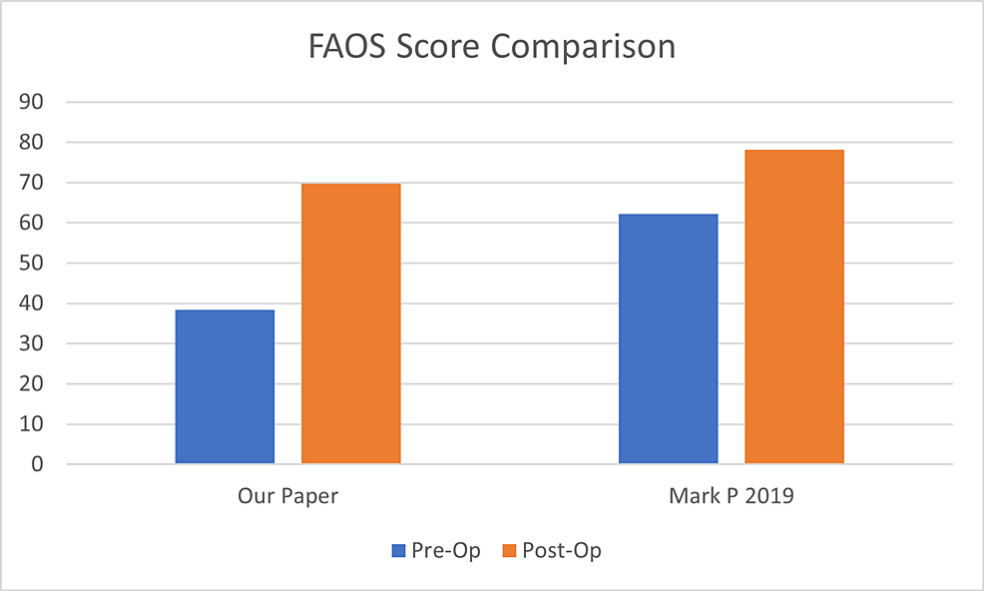
FAOS score comparison FAOS: Foot and Ankle Outcome Score

The pre-operative mean FAOS (Table 3) was 38.4 (minimum 14.3, maximum 60.1), and the post-operative mean FAOS (Table 3) was 69.7. The pre-operative FAOS was reported in one other study (Figure 6), with 25 patients treated with open repair [16]. The average FAOS was 62.2. The average post-operative FAOS reported in this study was 78.2. This corresponds closely to the results collected in our study.

**Table 3:** Pre-operative and post-operative FAOS numbers broken down into specific components and associated P-values. FAOS: Foot and Ankle Outcome Score

FAOS	Pre-operative FAOS	Post-operative FAOS	P-value
Symptoms	15.9	7.7	<0.001
Pain	21.5	10.6	<0.001
Function, daily living	34.6	15.3	<0.001
Function, sports, and recreational activities	15.7	8.3	<0.001
Quality of life	10.0	4.9	<0.001

Of the 42 participants, 40 had no recurrent episodes of ankle sprain between their operation and our follow-up 2-4 years post-surgery. The recurrent ankle sprains suffered by two patients were exercise-induced and did not require revision surgery. Post-operatively, patients were also asked to rate their satisfaction, with 50% reporting they were very satisfied and the other 50% being satisfied. None of the patients reported they were dissatisfied with the surgery or expressed regret in undergoing the operation.

## Discussion

The gold-standard surgery for chronic lateral ankle instability has been the MBG due to its safe and reproducible techniques. It has been shown to produce successful results and is reliable for achieving ankle stability. In recent years, there has been a progressive inclination toward minimally invasive options to reduce long surgery durations, as well as the surgical curved incision, in an effort to reduce soft tissue trauma [17]. Arthroscopic surgery has produced superior results regarding cosmesis and the benefit of the diagnosis and treatment of intra-articular pathologies associated with chronic ankle instability [17-19].

Although the option of the arthroscopic approach has become available and has been shown to have added benefits, it is unsuitable for all patient types. Instances in which arthroscopic repair would not be suitable are revision surgeries, in the presence of significant scarring, where eczema is present, and when the ATFL is deemed unsuitable for repair, requiring the use of the retinaculum for plication to reinforce the ankle. This is where our modified approach differs from the traditional surgery, with the main difference being a smaller incision (1.5 cm) instead of the conventional 6-7 cm incision. The smaller incision allows for better cosmesis, greater access than an arthroscopic approach, better wound healing, and comparable outcomes to traditional MBG concerning aspects of pain, functional outcome, and stability as measured using VAS scores, Karlsson scores, and the FAOS.

Pain

This paper suggests that chronic ankle instability repaired through the MMBG is associated with an overall improvement in pain levels and the grading of symptoms, including instability, swelling, and gait abnormalities. We found that the MMBG decreased pain level post-operatively, based on the VAS pain score, and in multiple components of other scoring systems, such as the pain components of the Karlsson score and the FAOS. The VAS score was reported in five other studies, with 164 patients treated with open repair [9-13]. The average VAS scores in these studies decreased from 5.89 to 1.53. This corresponds closely to the results collected in our study. Our study also showed that the MMBG improved the Karlsson pain score from 7.98 to 18.33. Other components that might be associated with pain include stair climbing and running ability, the scores of which also showed improvements, indicating an increased ability to perform such functions.

Stability

The results of this study show that the MMBG is capable of achieving stability in an unstable ankle. Instability decreased post-operatively, as suggested by the improvement of the Karlsson score, from 4.8 to 14.2 under the component of instability. Patients also reported a reduced instability symptoms, such as swelling, grinding, clicking, and catching, as well as a reduced range of motion, as shown by the FAOS. Mark P. conducted a randomized controlled trial to compare the MBG and ligament augmentation reconstruction system (LARS) outcomes. In his trial, 47 patients participated, of which 22 were treated with LARS, and 25 were treated with MBG. At 1-year post-operation, both groups improved their FAOS scores, with the LARS group improving from 66.9 to 92.4 and the MBG group improving from 62.2 to 78.2 [16]. While their study reported higher total scores 1-yr post-operation for both the LARS and MBG groups compared to our study (1-yr post-operative FAOS Score 69.7), it is important to note that their baseline FAOS scores (pre-operative LARS FAOS: 66.9; pre-operative MBG FAOS: 62.2) were significantly higher than those reported in our study (pre-operative FAOS: 38.4). Thus, our study reports a greater degree of improvement in terms of FAOS score (38.3 to 69.7). It is crucial to note that Mark P's small sample size of 25 patients who underwent the MBG may not provide the most representative picture.

Furthermore, of the 42 participants, 40 had no recurrent episodes of ankle sprain between their operation and our follow-up 2-4 years post-surgery. The recurrent ankle sprains suffered by two patients were exercise-induced and did not require revision surgery. Our study shows that the stability achieved by the MMBG is comparable with other surgical methods, as reported in other studies.

Functional outcomes

Functional outcomes have also been shown to have improved compared with MBG. This is likely due to the increased stability gained from the surgery, the reduced pain due to the smaller surgical incision, and the reduced injury to soft tissue structures during surgery, leading to a shorter recovery time. Analysis of the FAOS suggests that function in daily activities, sports, and recreational activity improved post-operatively. The score for daily activities improved from 49.10 to 77.49, and the score for sports and recreational activities improved from 20 to 55. Patients also expressed that their quality of life improved, as they were less aware of their ankle condition and less troubled by their ankle. While the MMBG has shown improvements in pain reduction and ankle stability, a key contributor to overall long-lasting functional outcomes is the patient's compliance with post-operative rehabilitation. Cho et al. found that at intermediate follow-up (2 years) following MBG, the peroneal strength was restored to 82.6% compared to the unaffected ankle, and patient-reported function in daily and sports were satisfactorily improved [20]. DiGiovanni et al. and Pearce CJ et al. stressed that during the rehabilitation program following a lateral ligament injury or surgery for chronic ankle instability (such as the MBG), restoring ankle stability through strengthening exercises for the peroneal muscles is considered an important factor in achieving successful outcomes [21,22]. Thus, great emphasis must be placed on the patient's rehabilitation program and compliance to optimize functional outcomes after the surgery.

Strengths and limitations

Due to the recruitment method of this study, where participants were recruited from a single surgeon from a particular public hospital in Singapore, there was a low number of only forty-two. Recruitment from a single surgeon could also potentially lead to selection bias. This study also does not address the potential impact of surgeon expertise and experience. Undoubtedly, it is important to acknowledge that surgeon expertise and skills built from experience would affect surgical outcomes, including variable post-op stability and pain.

## Conclusions

In summary, this study confirms the hypothesis that the MBG can achieve comparable results compared to the traditional MBG while providing increased patient suitability compared to the Arthroscopic Brostrom Gould surgery. This study has strongly suggested that MBG is an effective alternative to the arthroscopic and traditional MBG that helps with a more customized and suitable treatment plan for patients.
